# Quantification of Sterol and Triterpenol Biomarkers in Sediments of the Cananéia-Iguape Estuarine-Lagoonal System (Brazil) by UHPLC-MS/MS

**DOI:** 10.1155/2016/8361375

**Published:** 2016-03-21

**Authors:** Giovana Anceski Bataglion, Hector Henrique Ferreira Koolen, Rolf Roland Weber, Marcos Nogueira Eberlin

**Affiliations:** ^1^Department of Chemistry, Federal University of Amazonas (UFAM), 69077-000 Manaus, AM, Brazil; ^2^ThoMSon Mass Spectrometry Laboratory, Institute of Chemistry, University of Campinas (UNICAMP), 13083-970 Campinas, SP, Brazil; ^3^DeMpSter Mass Spectrometry Group, Amazonas State University (UEA), 69050-010 Manaus, AM, Brazil; ^4^Marine Organic Chemistry Laboratory, Oceanography Institute, University of São Paulo (USP), 05508-120 São Paulo, SP, Brazil

## Abstract

Sterols and triterpenols present in sedimentary cores from 12 stations along the Cananéia-Iguape estuarine-lagoonal system were investigated by ultra-high performance liquid chromatography tandem mass spectrometry (UHPLC-MS/MS). Ten sterols and three triterpenols were identified and quantified, indicating both natural and anthropogenic sources. The relative distributions of sterol and triterpenol showed that the study area is submitted to organic matter (OM) from the Ribeira de Iguape River, seawater, surrounding vegetation, and plankton production. The contribution of these sources depends on the region of the estuarine-lagoonal system and the depth of sediment. Regarding anthropogenic sources, only the samples submitted to freshwater flow from the Ribeira de Iguape River presented concentration of coprostanol higher than the threshold value and diagnostic ratios, coprostanol/(coprostanol + cholestanol) and coprostanol/cholesterol, that indicate moderate contamination by domestic sewage in that area of the estuarine-lagoonal system. Therefore, the approach used herein identified the OM sources and its transport along the Cananéia-Iguape estuarine-lagoonal system (Brazil), which is a complex of lagoonal channels located in a United Nations Educational, Scientific and Cultural Organization (UNESCO) Biosphere Reserve.

## 1. Introduction

Sterols, also known as alcohol steroids, are hydrophobic compounds that occur naturally in most of the living forms [1]. This class of compounds can be easily incorporated into sediments, where they remain well preserved, representing good tracers of sources, changes, and preservation of organic matter (OM) [2]. Sterols have been used to assess the OM origin in several estuarine systems around the world because of their recalcitrance and specific-source, which allows classifying the OM input from different sources, including terrestrial plants [3], phytoplankton [4], bacteria [5], and heterotrophic eukaryote and benthic algae [6]. Furthermore, besides natural OM sources, sterols have been applied in environmental studies to track human activities in coastal areas [7, 8].

Phytosterols, sterols C_28_ and C_29_, comprise the major sterol type found in estuarine sediments [9]. Campesterol, *β*-sitosterol, stigmasterol, and stigmastanol are plant sterols; thus they are used as geochemical biomarkers to assess the plant-derived OM input to aquatic systems [9, 10]. Algae often display predominance of C_27_ sterols (mainly cholesterol), with compositions different from those observed for vascular plants [11]. In addition, brassicasterol is frequently found in several algal classes, whereas cholestanol often occurs in the dinoflagellates class [11]. Other sterols, such as dinosterol and ergosterol, are exclusive biomarkers of OM from diatoms and fungi, respectively [12].

There are also important sterol biomarkers for anthropogenic OM, which are known as fecal sterols (e.g., coprostanol and epicoprostanol) and indicate contamination from domestic sewage [13]. Some authors point that absolute concentration of coprostanol should not be used as a unique and conclusive parameter to assess sewage contamination because of the possibility of* in situ* production. To solve this issue and to evaluate the degree of contamination by domestic sewage input, some ratios, such as coprostanol/(coprostanol + cholestanol) and coprostanol/cholesterol, have been proposed [14]. Higher plants, especially angiosperms, display triterpenols as typical and exclusive biomarkers. Triterpenols, such as lupeol and *α*- and *β*-amyrin, are the most common triterpenols found in sediments of riverine and estuarine environments [15].

Estuarine systems are essential compartments involved in the OM recycling and global carbon cycle [16]. Understanding the flows of OM from marine and rivers to estuaries provides information about its cycle in both water column and sediments [17]. Once OM in these systems constitutes a complex mixture of compounds from different sources, a reliable technique is required to overcome this complexity [18]. Sterols in sediment samples are generally analyzed by gas chromatography-mass spectrometry (GC-MS), which requires a cleanup step using alumina and silica columns, where sterols are separated from other classes of compounds. The fraction containing sterols is usually derivatized to increase their volatility and decrease the limits of detection (LOD) [19]. In this sense, liquid chromatography (LC) as a separation technique and tandem mass spectrometry (MS/MS) as a detection method are combined to overcome the inherent challenges of OM complexity [15]. Some methods employing analytical platforms based on liquid chromatograph tandem mass spectrometry (LC-MS/MS) for the analysis of selected sterols have been described over the last years for a range of matrices [20, 21] and more recently for sediments [15].

Information on sources, transport, and deposition of OM is scarce for the Cananéia-Iguape estuarine-lagoonal system, which is an important Brazilian ecosystem from the World Heritage List under natural criteria (UNESCO) due to extensive areas of mangrove and Atlantic forest. Consequently, this study aimed to determine the sterol and triterpenol composition and assess the OM origin in sedimentary cores along the study area. Based on that, an initial insight into the geochemistry and flow of OM in this estuarine-lagoonal environment was achieved.

## 2. Experimental

### 2.1. Study Area and Sampling

**Figure 1:**
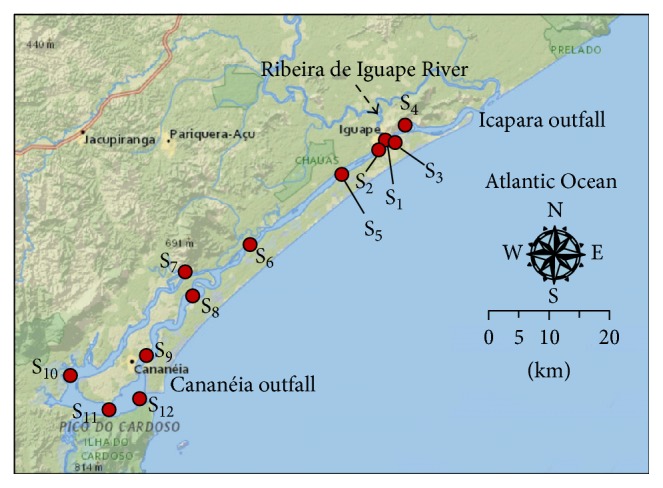
Map of study area showing the 12 sampling stations along the Cananéia-Iguape estuarine-lagoonal system, located in São Paulo state (Southeast Brazil). Content may not reflect National Geographic's current map policy. Sources: National Geographic, Esri, DeLorme, HERE, UNEP-WCMC, USGS, NASA, ESA, METI, NRCAN, GEBCO, NOAA, and Increment P Corp.

The Cananéia-Iguape estuarine-lagoonal system (Figure 1) is located in the Southeast Region of Brazil (São Paulo state). The opening of a 4 km long artificial channel linking the Ribeira de Iguape River to the Cananéia-Iguape estuarine-lagoonal system in the XIX century caused an enormous impact in this environment. After its opening, the channel eroded laterally reaching a width and depth of approximately 250 and 7 m, respectively. Thus, the channel was renamed to Valo Grande (“Big Scour”) in reference to the dimensions that it acquired. As a result of this erosion, environmental changes in the Cananéia-Iguape estuarine-lagoonal system were intensified, since about 60% of the main flow of the Ribeira de Iguape River was transferred to the system [22]. The affected area is an important ecosystem from the World Heritage List located between two cities (Cananéia and Iguape) with a protected green area consisting of typical vegetation of salt marshes, Atlantic forest, and mangroves [22].

A total of 60 samples from 12 collection sites (0–10 cm depth) were collected at the Cananéia-Iguape estuarine-lagoonal system. Sampling sites with their geographical locations and lithological characteristics are shown in Table 1. Sedimentary core samples were collected using a Rossfelder VT-1 vibracorer and stored at 4°C during transportation to the laboratory. Prior to the laboratory work, all sediment samples were freeze-dried, pulverized in a mortar, and sieved with a stainless steel sieve (<250 *μ*m) and stored at 4°C before analysis.

### 2.2. Chemicals and Reagents

(3*β*)-Cholest-5-en-3-ol (cholesterol, ≥99% purity), (3*β*,5*α*)-cholestan-3-ol (cholestanol, >95% purity), (3*β*,5*β*)-cholestan-3-ol (coprostanol, >95% purity), (3*α*,5*β*)-cholestan-3-ol (epicoprostanol, >95% purity), (3*β*,22*E*)-ergosta-5,7,22-trien-3-ol (ergosterol, >95% purity), (3*β*,22*E*)-ergosta-5,22-dien-3-ol (brassicasterol, >95% purity), (3*β*,24*R*)-ergost-5-en-3-ol (campesterol, >65% purity), (3*β*)-stigmast-5-en-3-ol (*β*-sitosterol, >98% purity), (3*β*,22*E*)-stigmasta-5,22-dien-3-ol (stigmasterol, >95% purity), (3*β*,5*α*)-stigmastan-3-ol (stigmastanol, >95% purity), and (3*β*)-lup-20(29)-en-3-ol (lupeol, ≥94% purity) were purchased from Sigma-Aldrich (St. Louis, MO, USA). The *α*-amyrin ((3*β*)-urs-12-en-3-ol) and *β*-amyrin ((3*β*)-olean-12-en-3-ol) were isolated [23] and used only to determine their retention times and MRM transitions. Based on the structural similarities between all of the sterols and their retention times, cholesterol-*d*_6_ (>98% purity) was used as internal standard (IS) for quantification of all of the compounds. HPLC-grade ethanol, methanol,* n*-hexane, and dichloromethane were obtained from J.T.Baker (Mexico City, DF, Mexico), and the water was purified by a Milli-Q gradient system (Millipore, Milford, MA, USA).

### 2.3. Sediment Extractions

The freeze-dried sediment samples were macerated, and ca. 20 g was spiked with IS solution to obtain a final concentration of 500 ng/mL, and they were then extracted with 50 mL of* n*-hexane and dichloromethane (1 : 1, v/v). The extraction was performed using a microwave system, MarsX (CEM, Mathews, NC, USA), operating at 1600 W with pressure and temperature gradients reaching 200 psi and 85°C in 5 min, respectively. The system was kept isothermal during 15 min, and then it returned to the room pressure and temperature conditions. The organic extracts were evaporated to 1 mL, and an aliquot of 50 *μ*L was diluted to exactly 1000 *μ*L of methanol.

### 2.4. Instrumentation and Chromatographic Conditions

Analysis of the sterols and triterpenols in the sedimentary OM extracts was conducted using an ultra-high performance liquid chromatography tandem mass spectrometry (UHPLC-MS/MS) system model LCMS-8040 (Shimadzu, Kyoto, Japan). The mass spectrometer system consists of a triple quadrupole equipped with an atmospheric pressure chemical ionization (APCI) probe. The chromatographic separation was performed on a Shimpack XR-ODS 2.2 *μ*m, 2.0 mm i.d., and 150 mm column (Shimadzu, Kyoto, Japan). The gradient elution at 30°C was as follows: 0–2 min (90% B), 2–8 min (100% B), and 8-9 min (90% B) at a flow rate of 0.6 mL/min. Solvent A was water, and solvent B was methanol.

APCI source was operated in the positive ion mode with the parameters as the following: corona current, 4.0 *μ*A; heat block temperature, 300°C; desolvation line temperature, 250°C. The precursor ions were determined in full scan experiments and the product ions were chosen by MS/MS experiments, and then the analyses were conducted by multiple ion monitoring (MRM) using two transitions for each standard compound (Table S1 in Supplementary Material available online at http://dx.doi.org/10.1155/2016/8361375), dwell time of 20 ms, and argon gas (99,996% purity from White Martins, São Paulo, SP, Brazil) pressure of 224 kPa, and no drying gas flow was used. Data were acquired and processed by the LabSolutions software version 5.53 SP2 (Shimadzu, Kyoto, Japan).

## 3. Results and Discussion

### 3.1. Quantitative Determination of Sterol and Triterpenol Biomarkers by UHPLC-MS/MS

**Figure 2:**
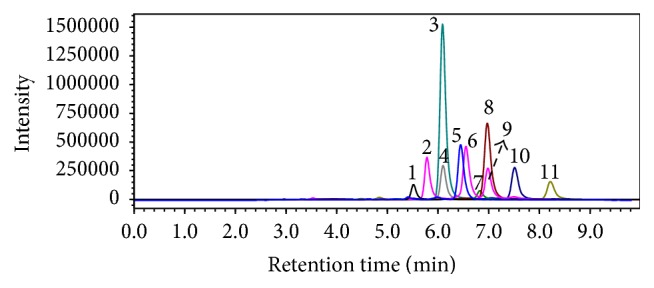
Representative UHPLC-MS/MS chromatogram for the analytes in a standard solution mixture (20 ng/mL). Ergosterol (1), epicoprostanol (2), lupeol (3), brassicasterol (4), cholesterol (5), coprostanol (6), campesterol (7), *β*-sitosterol (8), cholestanol (9), stigmasterol (10), and stigmastanol (11).

The UHPLC-MS/MS method using APCI provided high sensitivity and selectivity and required no derivatization of sterols and triterpenols. The most abundant signal in the full scan spectra corresponded to the neutral loss of water from the protonated sterol, which is consistent with previous studies [20, 21], and was chosen as precursor ion. The two most abundant signals in the MS/MS spectra were chosen as the product ions. The UHPLC-MS/MS method for determination of sterols and triterpenols was previously validated in relation to linearity, intraday and interday assays of accuracy and precision, and recovery [15]. A representative UHPLC-MS/MS chromatogram for the analytes in a standard solution mixture is shown in Figure 2.

Figure 2 shows the total ion chromatograms (TIC) for the compounds, which are the sum of the quantification and confirmation transitions. Peaks 2, 6, and 9 were properly separated corresponding to the sequential elution of the isomers epicoprostanol, coprostanol, and cholestanol. Peak 3 corresponds to the elution of lupeol, but its transitions also correspond to *α*- and *β*-amyrin, which present different retention times and were quantified using the calibration curve for lupeol. More details about the validation of the UHPLC-MS/MS method are described by Bataglion et al. [15].

### 3.2. Total Content of Sterol and Triterpenol Biomarkers

The validated UHPLC-MS/MS method was then applied to quantify sterols and triterpenols in 12 sedimentary cores from the Cananéia-Iguape estuarine-lagoonal system.  Table 2 lists the total concentrations of the sterols and triterpenols in the sedimentary core samples collected along the Cananéia-Iguape estuarine-lagoonal system.

The total concentrations of sterols and triterpenols along the sedimentary core samples cover a wide range, from 140 to 2784 ng/g dry weight. This significant variability may be associated with both lithological features of each sedimentary core and temporal changes in primary productivity or input of allochthonous OM. The total concentrations of sterols and triterpenols showed similar tendency along sedimentary cores 1, 2, and 3, which were collected closer to the Ribeira de Iguape River. These samples are characterized by a mixture of silt, clay, and fine sand rich in OM, which is due to the input of materials from the Ribeira de Iguape River. Although samples 4 and 5 are dominated by sand particles, the total concentrations of sterols and triterpenols were similar to those observed for the samples near to the Ribeira de Iguape River. Located in the middle of the estuarine-lagoonal system, samples 6 and 7 are predominantly sandy and were the ones with higher total concentrations of sterols and triterpenols at 0–2 cm depth. Also located in the middle of the estuarine-lagoonal system, sample 8 is completely constituted by sand particles in the first 4 cm, where the amount of fine particles (silt and clay) increases. These lithological features can be associated with the smallest and the highest total concentration at 0–2 cm and 8–10 cm, respectively. Located in the southern sector, samples 9, 10, 11, and 12 present a mixture of silt, clay, and thin sand, with samples 9 and 12 being the most sandy as a consequence of their proximity to the sea. Sample 10 showed the lower variability along the sedimentary core, which can be related to its location not affected by the inflow of seawater to the estuarine-lagoon system. In this group of samples, 9 and 12 were the ones with the lowest total concentrations of sterols and triterpenols along the sedimentary cores because of both the higher proportion of sand particles and influence of seawater inflow.

### 3.3. Profile of Sterol and Triterpenol Biomarkers

**Figure 3:**
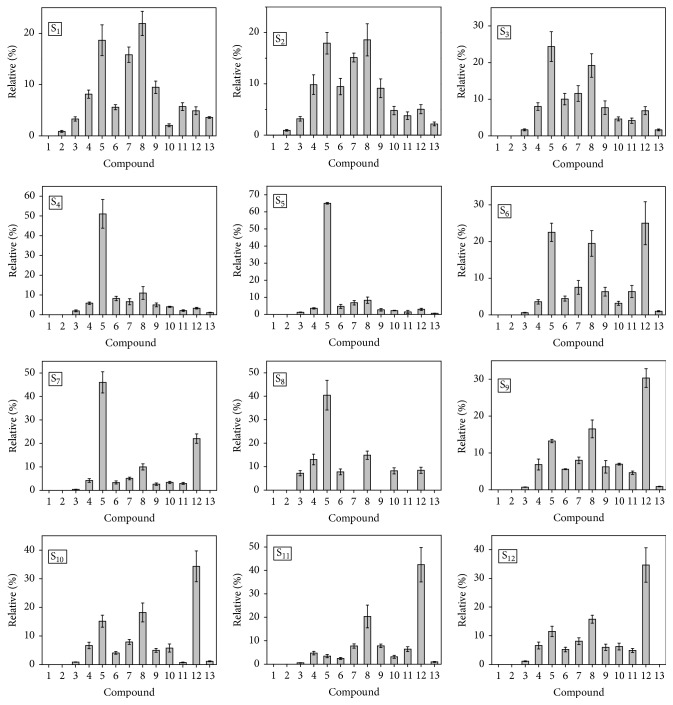
Relative distribution of sterols and triterpenols in sedimentary core samples at 0–2 cm from Cananéia-Iguape estuarine-lagoonal system, as revealed by UHPLC-MS/MS. (1) Ergosterol, (2) epicoprostanol, (3) coprostanol, (4) cholestanol, (5) cholesterol, (6) campesterol, (7) stigmasterol, (8) *β*-sitosterol, (9) stigmastanol, (10) brassicasterol, (11) lupeol, (12) *β*-amyrin, and (13) *α*-amyrin.

Figure 3 shows that the relative distribution of sterol and triterpenol biomarkers along the Cananéia-Iguape estuarine-lagoonal system presents a typical profile for each specific region. Sedimentary cores collected at stations 1, 2, and 3 (0–2 cm) present similar relative distributions of sterols and triterpenols, with cholesterol and *β*-sitosterol as the major compounds. These sterols are representative of different OM sources; *β*-sitosterol is one of the major sterols found in higher plants, whereas cholesterol is generally attributed to both zooplankton and phytoplankton (autochthonous OM) [24–26]. Although *β*-sitosterol is used as a biomarker of terrestrial OM in several geochemical studies, some authors also suggest the possibility of its origin from phytoplankton or even aquatic macrophytes [24, 27]. The relative contribution of these sources depends on both the studied environment and influence of such different OM sources. On the other hand, triterpenols, such as *α*-amyrin, *β*-amyrin, and lupeol, are considered highly specific geochemical biomarkers for higher plants [24, 28]. The three stations located near to the Ribeira de Iguape River are submitted to a high input of freshwater containing both terrestrial OM and nutrients. Therefore, stations 1, 2, and 3 are under influence of OM from higher plants, aquatic plants, and also plankton production, which justify the variety of compounds in the profiles of Figure 3.

The relative distribution of sterol and triterpenol biomarkers for the sedimentary cores collected at stations 4 and 5 (0–2 cm) was dominated by cholesterol, indicating accentuated input of OM from aquatic organisms to these stations. This region is under influence of freshwater from the Ribeira de Iguape River, but also of seawater that enters in the estuarine-lagoonal system by two outfalls (Cananéia and Icapara, Figure 1). Thus, the higher proportion of cholesterol in stations 4 and 5 is a result of the contribution of OM present in the seawater, which commonly presents it as the major sterol. The sedimentary cores collected at stations 6, 7, and 8 also present high contribution of cholesterol; however a significant proportion of *β*-amyrin can be observed, mainly for stations 6 and 7.

For all of the samples collected near to Cananéia Island, stations 9, 10, 11, and 12 (0–2 cm), the triterpenol *β*-amyrin was observed as the main compound, indicating predominance of OM from higher plants. Indeed, the Cananéia region is characterized by intense surrounding vegetation, mainly mangrove forest around the channels [22]. High contribution of the triterpenol *β*-amyrin was already observed for estuaries, where the mangrove vegetation,* Rhizophora mangle* (red mangrove) and* Avicennia germinans* (black mangrove), is dominant [29].

The relative distributions of sterol and triterpenol biomarkers for deeper samples (4–10 cm) of stations 1, 2, and 3 present similar profiles to those shown for samples 0–2 cm, but with a slight increase of *β*-sitosterol over cholesterol. This result may be associated with an increase of the planktonic production as a result of the high input of freshwater rich in nutrients from the Ribeira de Iguape River. Although the relative contribution of *β*-sitosterol was also higher for deeper samples of sedimentary core collected at station 4, cholesterol was always their major sterol. This slighter change indicates that station 4 is weakly submitted to freshwater from the Ribeira de Iguape River. An opposite situation occurs at station 5, where the relative distributions of sterol and triterpenol biomarkers for deeper samples (4–10 cm) become more similar to those obtained for stations near to the Ribeira de Iguape River. For stations 6, 7, and 8, a decrease of the relative contribution of cholesterol is observed for deeper samples, and the sterol and triterpenol distributions become more similar to those obtained for sedimentary core samples collected at Cananéia Island. For these ones, *β*-amyrin and *β*-sitosterol were the compounds with higher relative proportion along all the sedimentary cores.

Based on the relative distributions of sterol and triterpenol biomarkers, sediments from the Cananéia-Iguape estuarine system are submitted to OM from the Ribeira de Iguape River, seawater, surrounding vegetation, and plankton production. The relative contribution of these sources depends on the region of the estuarine-lagoonal system and the depth of sediment. In this sense, stations of the north region (1, 2, and 3) are more submitted to OM from the Ribeira de Iguape River, whereas samples 4 and 5 are submitted to both OM from the Ribeira de Iguape River and seawater, with different contribution along the sedimentary cores. Stations of the south region near Cananéia Island (9, 10, 11, and 12) are more submitted to OM from surrounding vegetation (mangrove), which dominates in all depths. In addition, stations located in the passage of seawater, such as 6 and 8, are submitted to OM from surrounding vegetation (mangrove) and seawater, which presents more contribution in the first cm of the sedimentary cores.

### 3.4. Contamination by Human Activities

In addition to evaluating the profiles of sterols and triterpenols in the sedimentary core samples from the Cananéia-Iguape estuarine-lagoonal system, we were also interested in assessing the levels of contamination by sewage discharge in this environment. Fecal sterols, coprostanol and epicoprostanol, were detected in 30 and 10 among the 60 samples analyzed, respectively. Coprostanol concentration ranged from 10 to 44 ng/g, whereas epicoprostanol ranged from 11 to 13 ng/g. The sedimentary core samples collected near to the Ribeira de Iguape River were the ones with the highest concentrations of coprostanol, which is a consequence of the input of domestic sewage from Iguape city that has a permanent population of 30,259 inhabitants. The lowest concentrations of coprostanol for sedimentary core samples collected near to Cananéia city may be associated with its lower permanent population of 12,601 inhabitants.

For comparative purposes, concentrations of coprostanol obtained for the Cananéia-Iguape estuarine system were compared to other estuarine and coastal environments in Brazil. The highest concentration of coprostanol found herein (44 ng/g for station 1 and depth of 0–2 cm) is much lower than those found in the Babitonga Bay that has a permanent population of 620,000 inhabitants (4040 ng/g) [3], in the inner shelf adjacent to Sergipe River that crosses Aracaju city with a population of 2 million inhabitants (184 ng/g) [16], and in the Itajaí-Açu estuary (8930 ng/g) that has a population of 200,000 inhabitants in Itajaí city and about 1.5 million in the mesoregion [30]. However, the highest concentration of coprostanol found herein is similar to that found for Santos estuary that is near to a city with ca. 420,000 inhabitants (48 ng/g) [15].

**Figure 4:**
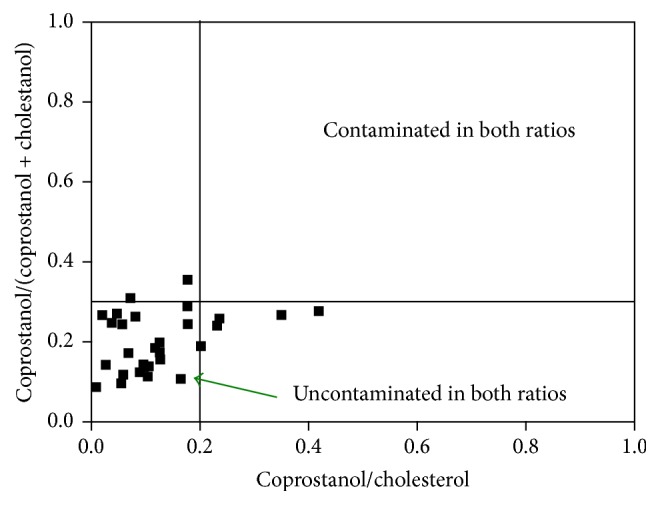
Plot of coprostanol/(coprostanol + cholestanol) ratios against coprostanol/cholesterol ratios for all sedimentary core samples that presented quantifiable concentration of these sterols. The lines inside the graph represent the regions that are considered contaminated or uncontaminated by the diagnostic ratios.

The concentration of 10 ng/g is considered a threshold value for sediments from natural coastal environments [14]. In addition, concentration higher than 500 ng/g is considered indicative of accentuated contamination by domestic sewage [14, 30]. The concentration levels of coprostanol observed herein (10 to 44 ng/g) are similar to or slightly higher than the threshold value (10 ng/g). As there is no consensus regarding the coprostanol concentration that is exclusively from domestic sewage, diagnostic ratios are also used to enhance the reliability of the assessment of contamination [31, 32]. In this sense, coprostanol/(coprostanol + cholestanol) and coprostanol/cholesterol ratios were calculated to evaluate the level of sewage contamination in the sedimentary core samples from Cananéia-Iguape system. Based on these ratios, sewage influence is not evident for the majority of sediment samples from the Cananéia-Iguape estuarine-lagoonal system (Figure 4), which presented coprostanol/cholesterol values that were <0.2 and coprostanol/(coprostanol + cholestanol) values that were <0.3 [31, 32]. The samples that fall in the region considered contaminated in one of the ratios correspond mainly to sedimentary core collected at station 1, which is submitted to the OM coming from the Ribeira de Iguape River.

Although some samples presented concentration of coprostanol higher than the threshold value and diagnostic ratios that indicate contamination by domestic sewage to the estuarine system, these results suggest a moderate input once concentration of coprostanol higher than 500 ng/g and coprostanol/(coprostanol + cholestanol) ratio higher than 0.7 are found for environments submitted to accentuated input.

## 4. Conclusions

In summary, this is the first study that assessed sterol and triterpenol biomarkers in sedimentary cores of the Cananéia-Iguape estuarine-lagoonal system. Typical distribution of sterols and triterpenols was observed for each region of the study area. The region near the Ribeira de Iguape River is mainly submitted to OM of its freshwater outflow, presenting biomarkers of higher plants, aquatic plants, and plankton production. As pointed by coprostanol/(coprostanol + cholestanol) and coprostanol/cholesterol ratios and the absolute concentrations of coprostanol, this area also presented a moderate sewage input as a consequence of the proximity to Iguape city. The south region is characterized by a strong input of OM from surrounding vegetation, mainly mangroves, as revealed by the high proportions of *β*-sitosterol and *β*-amyrin. At the same time, all regions submitted to seawater inflow present a higher contribution of cholesterol, mainly in the first cm. Therefore, the assessment of sterols and triterpenols by UHPLC-MS/MS gave us insights into source, transport, and deposition of OM in the Cananéia-Iguape estuarine-lagoonal system.

## Supplementary Material

Mass spectrometry parameters for the MRM transitions: Q1 voltage, Q3 voltage, and collision energy (CE), are shown in Table 1S in Supplementary Material.

## Figures and Tables

**Table 1:** Geographical locations and lithological characteristics for the 12 sampling sites in the Cananéia-Iguape estuarine-lagoonal system.

Station	Longitude (W)	Latitude (S)	Lithological characteristics
1	47°33′775′′	24°42′855′′	Similar proportion of sand, silt, and clay
2	47°33′711′′	24°43′180′′	Predominance of silt and clay
3	47°43′480′′	24°42′127′′	Predominance of silt and clay
4	47°51′943′′	24°42′022′′	Predominance of sand (>80%)
5	47°37′093′′	24°45′080′′	Predominance of sand (>70%)
6	47°45′093′′	24°49′080′′	Predominance of sand (>80%)
7	47°51′449′′	24°54′010′′	Predominance of sand (>90%)
8	47°52′071′′	24°57′723′′	100% of sand in the first 4 cm
9	47°55′168′′	25°01′020′′	Predominance of sand (>70%)
10	48°01′214′′	25°01′017′′	Similar proportion of sand, silt, and clay
11	47°58′047′′	25°04′088′′	Predominance of clay (50%)
12	47°55′693′′	25°04′278′′	Predominance of sand (>60%)

**Table 2:** Total concentrations of sterols and triterpenols (ng/g of dry weight) in sedimentary core samples from the Cananéia-Iguape estuarine-lagoonal system (*n* = 3).

Stations	0–2 cm	2–4 cm	4–6 cm	6–8 cm	8–10 cm
1	1337 ± 67	1076 ± 140	680 ± 75	873 ± 87	1265 ± 63
2	1335 ± 93	1315 ± 145	283 ± 25	585 ± 53	420 ± 34
3	1480 ± 74	1157 ± 116	679 ± 54	433 ± 30	466 ± 51
4	1023 ± 92	804 ± 88	420 ± 34	517 ± 52	669 ± 87
5	1796 ± 215	751 ± 53	1187 ± 117	309 ± 31	582 ± 35
6	2180 ± 239	899 ± 54	2429 ± 194	1556 ± 124	805 ± 80
7	2526 ± 152	1162 ± 93	916 ± 64	586 ± 41	378 ± 30
8	140 ± 8	293 ± 23	1912 ± 134	2272 ± 159	2784 ± 167
9	1377 ± 96	946 ± 57	677 ± 41	894 ± 54	1126 ± 79
10	1870 ± 205	1860 ± 93	1762 ± 141	2624 ± 210	2298 ± 161
11	1772 ± 195	1678 ± 134	1597 ± 143	1879 ± 169	1975 ± 158
12	911 ± 73	831 ± 58	430 ± 26	520 ± 36	795 ± 87
