# Supplementary material for: Quantification of Sterol and Triterpenol Biomarkers in Sediments of the Cananéia-Iguape Estuarine-Lagoonal System (Brazil) by UHPLC-MS/MS
Source: Int J Anal Chem. 2016 Mar 21;2016:8361375. doi: 10.1155/2016/8361375 (PMC4819115; doi:10.1155/2016/8361375)
Supplement: Supplementary file 1 — Mass spectrometry parameters for the MRM transitions: Q1 voltage, Q3 voltage, and collision energy (CE), are shown in Table 1S in Supplementary Material. [file 8361375.f1.pdf]

Table S1. Mass spectrometry parameters for MRM transitions: Q1 voltage, Q3 voltage and collision energy (CE)

Compound	Transitions	Q1 (V)	Q3(V)	CE (V)
cholesterol	369>95*	-17	-19	-32
	369>135	-25	-28	-22
epicoprostanol/coprostanol/cholestanol	371>81	-13	-17	-36
	371>95*	-17	-19	-29
ergosterol	379>69*	-30	-29	-26
	379>83	-30	-17	-24
brassicasterol	381>69*	-13	-28	-29
	381>135	-25	-28	-24
campesterol	383>81	-25	-16	-40
	383>161*	-25	-18	-21
stigmasterol	395>81*	-18	-16	-41
	395>107	-18	-22	-35
β-sitosterol	397>81	-14	-16	-41
	397>95*	-14	-19	-34
stigmastanol	399>95*	-14	-19	-33
	399>149	-14	-29	-22
lupeol/β-amyrin/α-amyrin	409>95*	-29	-19	-37
	409>137	-19	-16	-22

* transition of quantification
